# Progress in Bacteriology

**Published:** 1899-04-29

**Authors:** 


### PROGRESS IN BACTERIOLOGY.

**Tuberculosis.**—Levene[1] reviews the present day knowledge of the bio-chemistry of bacillus tuberculosis. Prudden first observed that the bodies of dead tubercle bacilli were capable of exciting lesions similar to those in ordinary tuberculosis. Buchner attributed this action to a proteid body, and Hoffman precipitated by means of alcohol a proteid from the watery extract of tubercle bacilli, and a nucleo-proteid has been isolated by Gileo-Galeotti. Levene's own experiments lead him to the conclusion that the bacilli contain no proteid of an albumin nature, but only nucleo-proteids. It is noteworthy that the coagulation point of one of these nucleo-proteids is identical with the sterilisation point of the tubercle bacillus, namely, about 70 deg. C. Ruppel[2] agrees that solutions of the bacilli give no marked albumin reactions, but contain atmid-albumoses, such as described by Neumeister. He also has isolated from such solutions three varieties of fats, and has demonstrated that the tubercle bacillus can act on albuminous solutions forming peptones and tryptophans.

**Human and Avian Tuberculosis.**[3]—The true relationship of human to avian tuberculosis is as yet unknown. There are marked cultural and experimental differences between the bacillus as found in man and in birds. That of birds is capable of growth at 43 deg. C., a temperature fatal to the organism of the human disease. Mammalian tuberculosis has never been communicated to birds by inoculation, and avian tubercle inoculated into mammals may, or may not, excite tuberculosis; thus, whilst the rabbit and horse are susceptible, the dog and guinea pig are refractory. Nocard claims to have established that these two apparently different bacilli are only two varieties of the same species. Human tubercle bacilli, enclosed in collodion capsules, are introduced into the peritoneal cavity of fowls. Here, nourished by the body fluids, and secure within the capsule from the attacks of phagocytes, they take on the character of avian tubercle bacilli, and after passage through one or more hosts, are capable of exciting true tuberculosis in birds.

**Tuberculosis in Fish.**[4]—Dubar, Bataillon, and Terre record an instance in which the spata and dejecta of a patient suffering from advanced tuberculosis were thrown into a pond causing the death of many of the fish, whose viscera were, on examination, found to be full of tubercle bacilli. Healthy fish fed experimentally with similar material also succumbed. Living at the lower temperature of the fishes' body the tubercle bacillus becomes so attenuated as to be incapable of infecting mammals when inoculated into them, so that there would appear to be little danger from the eating of diseased fishes. This is the first recorded instance of tuberculosis in cold-blooded animals.

**Tubercle Bacilli in Butter.**—Such widely different results have been obtained from the bacteriological examination of butter that it seems probable that there has been some confusion between the true bacillus and the pseudo-tubercle bacillus of Rabinowitch.[5] The last-named observer failed to find the true organism in 80 samples of butter examined at Berlin and Philadelphia, but found the pseudo-bacillus in about one-third of the samples. Subsequently, out of 15 specimens examined at Berlin the true bacillus was found once, and that in 70 per cent. of the produce of one factory. Obermüller, on the other hand, found the virulent bacilli in each of 14 samples; and Petri, 30 per cent. of true bacilli to 60 per cent. of the pseudo variety. Hormann and Morgenroth[6] obtained three positive results out of ten inoculation experiments, and conclude that butter can undoubtedly convey infection, and is a not uncommon medium. They regard the pseudo-bacillus as an altered type of the true organism. Their pseudo-bacillus stains as does the tubercle bacillus, and resembles it morphologically. It grows more readily, however, than does the latter, and forms indol in glycerine bouillon, and gives a precipitate with acids, two characters foreign to the other variety. A disagreeable ammoniacal smell and a coppery red growth are features peculiar to this pseudo-bacillus. It has been detected on the grass of certain pastures, and it is thought that cattle may thus become infected. As a remedy against possible infection, it is suggested that Pasteurised cream be used for butter making.

**Tubercle Bacilli in Milk.**—Hammond[7] recommends the following as a simple and rapid method of detecting tubercle bacilli in fluids such as milk. Add to the milk a 5 per cent. solution of glacial carbolic acid, so as to check the growth of any organisms present. Centrifugalise 15 cc. of the milk for 15 minutes, and treat the precipitate with 3 cc., of 5 per cent. solution of caustic potash for three minutes. Add distilled water to make up to 15 cc., and again centrifugalise for 20 minutes. The sediment, which has by the treatment been freed from fat and proteid matter is now ready for microscopical examination. It is claimed that by this method bacilli may be detected in fluids in which they are so sparsely scattered as to give negative results when inoculated into guinea pigs.

**Pseudo-Tuberculosis.**—Hayem's observations point to the fact that this disease, common in birds and rodents, may occur also in the human subject. Muir's[8] observations have reference more especially to the disease in birds, and he regards the bacillus isolated by him as resembling the variety described by Nocard. The disease occurs in epidemic form amongst caged singing birds, and is very fatal. The lesions are chiefly confined to the spleen and liver, which are studded with yellowish nodules, which on section are found to be studded with bacilli. These bacilli stain with aniline dyes, but not with Gram's method. They are easily decolourised. In length they are about 2$\mu$. Surrounding tissues are little affected. Growth is abundant on agar or gelatine, the organism being non-motile and devoid of spores. Inoculated into birds there results a local necrosis, general infection, and formation of nodules in liver and spleen. Infection may also be spread by feeding on pure cultures. Similar results were obtained upon rodents. Comparing the lesions with those of true tubercle they are seen to be more marked by leucocytic infiltration, with rapid caseation and softening. Giant cells are absent, and degenerative changes are generally advanced before proliferative changes.

**Antitoxins for Tuberculosis.**—Trudean and Baldwin[9] have conducted a series of researches extending over four years upon this subject. From the low degree of toxicity of tubercular products they anticipated that to produce and demonstrate antitoxic properties in serums would be a matter of much difficulty. To be of service an antituberculous serum should possess both antitoxic and germicidal properties. Their products of germ growth had the chemical reactions of albumoses and nucleoproteids. The following is a summary of their results: Killed thymus culture of tubercle bacilli injected into sheep conferred no power upon the serum. The serum of chickens inoculated intraperitoneally with mammalian tubercle bacilli in no way affected the course of disease in guinea-pigs. No curative, antitoxic, or germicidal power was found to exist in the blood of sheep injected with tuberculin. The injection of non-virulent bacilli in no way modified the serum, but if virulent bacilli were employed there was slight antitoxic value imparted. Of a variety of so-called antituberculous serums tested only one indicated antitoxic power; this, it was ascertained, was obtained from a horse inoculated with non-virulent cultures. None of serums prevented local or general reaction from small doses of tuberculin. In spite of these negative results the writers regard the outlook for an efficient tuberculosis antitoxin as by no means a hopeless one. Berlioz[0] claims to increase the resisting power of the human subject against the tubercle bacillus by the use of medicated serum. He employs ox serum, giving doses of 30 grams. twice a day per rectum. To the serum is added 1 per cent. of phosphite of guaiacol, together with organic extracts from liver, brain, spleen, and lungs. Under this treatment, which must be continued for weeks or months, the weight increases, nutrition improves, and strength is increased. There is diminution of the expectoration, and disappearance of the night sweats. Somewhat similar is Meyer[11] and Scoguamigli's treatment by glandulen, a substance prepared from the bronchial glands of sheep, by washing with alcohol, drying, freeing from fat, and pulverising. As the glands during life form the barrier to general infection, so it is thought their administration may assist the resisting power of the body. Clinically, the results of this treatment have in the hands of the authors been most satisfactory.

